# Randomized controlled pilot study of an educational video plus telecare for the early outpatient management of musculoskeletal pain among older emergency department patients

**DOI:** 10.1186/s13063-017-2403-8

**Published:** 2018-01-05

**Authors:** Timothy F. Platts-Mills, Allison G. Hollowell, Gary F. Burke, Sheryl Zimmerman, Joseph A. Dayaa, Benjamin R. Quigley, Montika Bush, Morris Weinberger, Mark A. Weaver

**Affiliations:** 1 Department of Emergency Medicine and Division of Geriatrics, Department of Medicine, University of North Carolina Chapel Hill, 101 Manning Drive, CB #7010, Chapel Hill, NC 27599-7010 USA; 2 School of Medicine, University of North Carolina at Chapel Hill, Chapel Hill, NC USA; 3 Department of Emergency Medicine, University of North Carolina at Chapel Hill, Chapel Hill, NC USA; 4 School of Social Work, University of North Carolina at Chapel Hill, Chapel Hill, NC USA; 5 Gillings School of Public Health, University of North Carolina at Chapel Hill, Chapel Hill, NC USA; 6 Department of Biostatistics, University of North Carolina at Chapel Hill, Chapel Hill, NC USA

**Keywords:** Musculoskeletal pain, Geriatrics, Emergency medicine, Pain

## Abstract

**Background:**

Musculoskeletal pain is a common reason for emergency department (ED) visits. Following discharge from the ED, patients, particularly older patients, often have difficulty controlling their pain and managing analgesic side effects. We conducted a pilot study of an educational video about pain management with and without follow-up telephone support for older adults presenting to the ED with musculoskeletal pain.

**Methods:**

ED patients aged 50 years and older with musculoskeletal pain were randomized to: (1) usual care, (2) a brief educational video only, or (3) a brief educational video plus a protocol-guided follow-up telephone call from a physician 48–72 hours after discharge (telecare). The primary outcome was the change from the average pain severity before the ED visit to the average pain severity during the past week assessed one month after the ED visit. Pain was assessed using a 0–10 numerical rating scale.

**Results:**

Of 75 patients randomized (mean age 64 years), 57 (76%) completed follow up at one month. Of the 18 patients lost to follow up, 12 (67%) had non-working phone numbers. Among patients randomized to the video (arms 2 and 3), 46/50 viewed the entire video; among the 25 patients randomized to the video plus telecare (arm 3), 23 were reached for telecare. Baseline pain scores for the usual care, video, and video plus telecare groups were 7.3, 7.1, and 7.5. At one month, pain scores were 5.8, 4.9, and 4.5, corresponding to average decreases in pain of -1.5, -2.2, and -3.0, respectively. In the pairwise comparison between intervention groups, the video plus telecare group had a 1.7-point (95% CI 1.2, 2.1) greater decrease in pain compared to usual care, and the video group had a 1.1-point (95% CI 0.6, 1.6) greater decrease in pain compared to usual care after adjustment for baseline pain, age, and gender. At one month, clinically important differences were also observed between the video plus telecare and usual care groups for analgesic side effects, ongoing opioid use, and physical function.

**Conclusion:**

Results of this pilot trial suggest the potential value of an educational video plus telecare to improve outcomes for older adults presenting to the ED with musculoskeletal pain. Changes to the protocol are identified to increase retention for assessment of outcomes.

**Trials registration:**

ClinicalTrials.gov, NCT02438384. Registered on 5 May 2015.

**Electronic supplementary material:**

The online version of this article (doi:10.1186/s13063-017-2403-8) contains supplementary material, which is available to authorized users.

## Background

Acute musculoskeletal pain is one of the most common reasons for emergency department (ED) visits by older adults [1–3]. Acute episodes of musculoskeletal pain can result from traumatic events (e.g., fall or motor vehicle collision), atraumatic conditions (e.g., acute episode of back or neck pain) or acute exacerbation of chronic pain conditions (e.g., osteoarthritis of the knee). Regardless of etiology, most ED patients with a primary complaint of musculoskeletal pain are discharged home [1, 4]. These ED visits are medically important for patients presenting with musculoskeletal pain for several reasons. First, an estimated 10–25% of patients transition from acute to chronic musculoskeletal pain following an ED visit [4, 5], and chronic musculoskeletal pain is the single largest cause of disability and reduced quality of life in the USA [6]. Second, analgesics prescribed in the ED frequently produce side effects and adverse events that result in medication discontinuation and return ED visits [7, 8]. Third, the initiation of opioids for acute musculoskeletal pain in the ED has been associated with long-term opioid use, placing patients at risk for opioid addiction and opioid overdose-related death [9].

Recognizing these concerns, emergency providers have the opportunity to improve outcomes for older adults with musculoskeletal pain by optimizing early outpatient treatment. Because most of these patients will make decisions regarding use, dosing, and discontinuation of medications without immediate access to a medical provider, patient self-management education is critical to achieving this goal. Given the risks of analgesics in older adults, behaviors that promote recovery, such as physical activity, sleep, and relaxation, are also likely to be valuable. In patients with chronic pain conditions such as arthritis, pain self-management education reduces pain and disability [10, 11]. Successful early outpatient treatment of acute musculoskeletal pain will likely also require close follow-up with a medical provider to review and adjust treatment regimens [12]. In patients with chronic pain, an iterative process of weekly phone calls to assess symptoms and guide therapy improves pain symptoms compared to usual care [13]. Despite the large number of ED visits for acute musculoskeletal pain and the importance of post-discharge pain management, most clinical trials have focused exclusively either on treatment provided in the ED or outpatient treatment of chronic pain [13–15]. We sought to address this gap by developing an educational video for older adults presenting to the ED with acute musculoskeletal pain, combined with a medical provider phone call 48–72 hours after the ED visit to support both pharmacologic and behavioral methods of pain management.

The purpose of this pilot study was twofold: (1) assess the feasibility of a clinical trial to test an educational video and pain management telecare intervention for older ED patients with acute musculoskeletal pain and (2) obtain preliminary estimates of the effect of the video plus telecare vs. usual care and of the video alone vs. usual care on pain symptoms and other outcomes over the first month.

## Methods

### Overview to study design and setting

We conducted a pilot randomized controlled trial of patients aged 50 years and older presenting to an academic ED in Southeastern USA with a chief complaint of musculoskeletal pain. The trial was registered at ClinicalTrials.gov (NCT02438384), and abides by Consolidated Standards of Reporting Trials guidelines. The study was approved by the site’s institutional review board. Recruitment took place during a period of 11 months between August 2015 and March 2017. Recruitment was interrupted for a total of 7 months during the winters of 2015 and 2016 and the summer of 2016 due to limited availability of research assistants (RAs). Screening and signed informed consent were completed by trained RAs. Patients, stratified by age (50–64 or ≥65 years), were randomized into one of three groups (described subsequently) using computer-generated, sequentially numbered opaque envelopes; randomization occurred following completion of baseline data collection. Patients were followed for 30 days.

### Participants

RAs approached all English-speaking patients aged 50 years and older presenting to the ED with musculoskeletal pain. RAs monitored the ED track board for chief complaints suggesting musculoskeletal pain (e.g., back pain, leg pain, fall). We excluded patients complaining of head, chest, or abdominal pain and patients with pain suspected to be due to infection, ischemia, or another non-musculoskeletal problem, such as a kidney stone. If RAs were unsure if the patient’s pain was musculoskeletal in nature, input was requested from the attending physician before approaching the patient. Patients were also excluded if they were a prisoner or on a psychiatric hold, were critically ill (Emergency Severity Index score of 1), or had moderate or severe cognitive impairment (Six-Item Screener score of 3 or less) [16].

### Measures

During baseline interviews, RAs obtained information on patient demographics, contact information, health and physical function, and pain characteristics. Health was assessed using a self-reported general health measure [17], number of patient-reported prescription medications taken daily [18], and the presence or absence of pain on a daily basis in their bones, muscles, tendons, ligaments, or joints in the past 6 months [19]. Capacity for physical function was assessed using self-reported ability to walk, climb stairs, and carry a bag, producing an overall score ranging from 0 to 12 [5, 20]. Pain was assessed in the ED by asking patients “Since your pain began, on average how intense has this pain been on a scale from 0-10?” (0 = “no pain” and 10 = “pain as severe as it could possibly be”).

Follow-up assessments occurred at 1 month after the ED visit, or for patients who were admitted, 1 month after discharge from the hospital. Pain was reassessed by asking patients to rate their average pain over the past week using the same 0–10 scale. Capacity for physical function was reassessed at 1 month. Participants were also asked if they had received any prescriptions or medication recommendations upon discharge from the ED, the names of the medications, how long they took them, and if they experienced any side effects from the medications. Patients were also asked if they had “taken any other pain medications other than those prescribed or recommended to you in the ER one month ago?” Side effects were assessed by asking patients to affirm their experience of any of the following side effects commonly experienced by older adults discharged after an ED visit for musculoskeletal pain: fatigue, drowsiness, trouble sleeping, trouble thinking, dizziness, unsteadiness, nausea, vomiting, constipation, abdominal pain, black or bloody stool, trouble urinating, loss of appetite, itching, or shortness of breath [7]. Additionally, participants were asked if they had experienced any side effects not included in the list. Healthcare utilization was assessed by asking patients if they had returned to an ED during the month following the ED visit or if they had seen any other doctor since their ED visit.

The primary outcome was the change in average pain from the average pain prior to the ED visit to the average pain during the week prior to the one-month assessment. Negative values reflect decreased pain severity. We also report the percentage of patients with at least a 1-point decrease in pain during this time period; a 1-point decrease has been described as the minimal clinically important difference in chronic musculoskeletal pain [21].

### Intervention

Participants were randomized to one of three intervention groups: (1) usual care, (2) viewing a brief interactive educational video in the ED, or (3) viewing the video and receiving a follow-up phone call from one of two study physicians who were not involved in the patient’s ED care. Patients in all three arms received prescriptions, recommendations for over-the-counter medications, and behaviors at the discretion of the ED provider without any restrictions imposed by the study.

#### Conceptual approach for the intervention

The development of the interventions was grounded in shared decision-making, a process in which both patients and providers contribute to the medical decisions. The outpatient treatment of musculoskeletal pain in older adults is an appropriate context for shared decision-making because (1) there are multiple possible treatment options; (2) there is uncertainty about the best approach; and (3) the optimal approach often depends on specific characteristics and values of the patient (e.g., pain tolerance, anticipated impact of pain on psychological health, risk of adverse events from specific medications, likelihood of participating in recovery-promoting behaviors). Patient engagement in the decision process in the ED also makes sense because most patients are discharged, and many make decisions about which medications to take, when to take them, and what behaviors to pursue without input from a medical provider. The feasibility and value of shared decision-making in the ED is well-established [22, 23]. Further, our research indicates that older ED patients with musculoskeletal pain want to be involved in pain treatment decisions and that shared decision-making is associated with improved outcomes when it occurs [24, 25].

#### Interactive video

We developed a 13-minute educational video that presented information on the pharmacologic and non-pharmacologic management of musculoskeletal pain [26]. The pharmacologic section described the common brand names, indications, contraindications, recommended dosage, and side effects of the most commonly given oral analgesics: acetaminophen, nonsteroidal anti-inflammatory drugs (NSAIDs), and opioids. The non-pharmacological pain management section described the importance of physical activity, sleep, social support, and relaxation. At the end of each section, a multiple-choice question was presented to the participant to promote interaction and reinforce learning. After the participant selected a response, they were then provided with the answer. The reading level for the video content was 8.6 based on the Flesch-Kincaid readability test. [27] Throughout the video, patients were reminded that people will have different pain management needs and that response to treatments and consideration of alternatives should be reviewed at frequent intervals with their provider.

#### Telecare

Telecare consisted of a phone call from an emergency physician 48–72 hours following discharge from the ED. For patients who were admitted to the hospital, this phone call occurred 48–72 hours after they were discharged from the hospital. During the phone call, the physician followed a standardized protocol that first determined whether the patient was likely to benefit from a conversation about pain management. Patients who reported experiencing a current pain rating of 4 or higher (indicating moderate or severe pain [28]), pain affecting their normal activities, pain affecting sleep, pain medication side effects, or who requested to talk to a physician about their pain then received further conversation about pain management. Patients meeting the criteria for further conversation were asked what pain medications they were currently taking and whether they were experiencing pain relief or side effects, and if they had any specific existing conditions that would be a contraindication for certain analgesics (e.g., medication allergy, liver disease, kidney disease, history of stomach ulcers or gastrointestinal bleeding). Using this information, the physician then followed a standardized protocol to review the patient’s goals and priorities for pain management, make suggestions about possible changes to pain management, and elicit feedback from patients (Additional file 1). The protocol was modified from a telecare protocol used for a large clinical trial to improve outpatient management of chronic pain [13] based on input from experts in pain care, emergency care, and risk communication. For those without scheduled follow up, patients were asked if they had health insurance and were referred to local clinics accordingly.

### Statistical analysis

Sociodemographic and health characteristics are presented for patients in each of the three study arms. Intention-to-treat (all randomized patients) and per-protocol analyses (participants who viewed the video to completion and received telecare follow up if randomized to receive those interventions) were performed. Change in average pain scores for each group was calculated. Changes in pain were then estimated with adjustment for age, gender, and baseline pain scores using the STATA command predxcat, which estimates values for each group using mean total sample values for variables used for adjustment. Pairwise differences in change in average pain between each of the three possible comparison pairs (video plus telecare vs. usual care, video alone vs. usual care, video plus telecare vs. video alone) were estimated along with 95% confidence intervals, adjusted for baseline pain, age, and gender. For the purpose of adjustment, baseline pain and age were treated as continuous variables. Process outcomes including analgesics prescribed or recommended by the emergency provider and opioid prescription by another provider after the ED visit are described. All analyses were performed using STATA 14.1 (StataCorp LP, College Station, TX, USA).

## Results

**Fig. 1:**
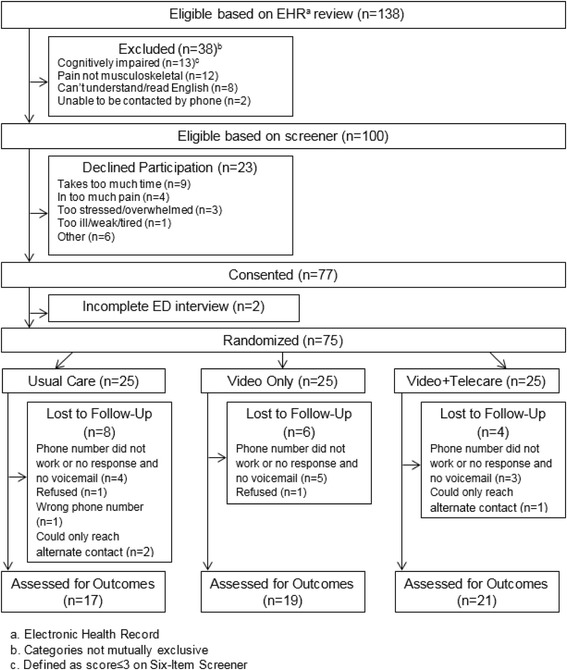
Flow diagram of the screening and enrollment process

Of 138 patients screened for eligibility, 100 (72%) were eligible and 77 (77%) gave consent (Fig. 1). Of eligible patients who declined participation, the most common reasons were “takes too much time” (*n* = 9), “in too much pain” (*n* = 4), and “too stressed/overwhelmed” (*n* = 3). Of the 77 consenting participants, two patients were discharged prior to randomization, leaving a final randomized sample of 75 patients and an enrollment rate of 7 patients per month.

Of the 75 participants, 66% were female and 75% were white. The majority of patients was community-dwelling (96%) and discharged from the ED following treatment for musculoskeletal pain (79%). Most patients (89%) reported moderate to severe pain (pain score ≥4) in the ED, and 60% received pain medication prior to the ED interview. The most common locations of pain were the leg (24 patients), back (19 patients), hip (15 patients), neck (6 patients), and arm (6 patients). Patient characteristics by study arm are presented in Table 1.

**Table 1:** Patient characteristics (*n* = 75)

Characteristic	Usual care (*n* = 25)	Video only (*n* = 25)	Video + telecare (*n* = 25)
Female, *n* (%)	18 (72)	19 (76)	13 (52)
Age, years			
Mean (SD)	66 (2.1)	63 (2.1)	61 (2.1)
50–64, *n* (%)	14 (56)	18 (72)	16 (64)
≥ 65, *n* (%)	11 (44)	7 (28)	9 (36)
Non-white, *n* (%)	5 (31)	6 (33)	8 (42)
Formal education, *n* (%)			
High school or less	11 (44)	14 (56)	9 (36)
> High school	14 (56)	11 (44)	16 (64)
Home, *n* (%)			
Private home	24 (96)	25 (100)	24 (96)
Assisted living	1 (4)	0 (0)	1 (4)
Daily medications^a^, mean (SD)	4.8 (0.8)	4.5 (0.8)	4.2 (0.8)
Self-reported health^b^, *n* (%)			
Good, very good, or excellent	20 (83)	20 (80)	19 (76)
Fair or poor	4 (17)	5 (20)	6 (24)
Chronic musculoskeletal pain^c^, *n* (%)	11 (73)	13 (77)	11 (61)
Pain due to injury, *n* (%)	10 (63)	12 (67)	13 (68)
Disposition^b^, n (%)			
Admitted	2 (8)	5 (20)	9 (36)
Discharged	22 (92)	20 (80)	16 (64)

^a^Defined as a numerical answer to the question “How many prescription medications do you take daily?”

^b^*N* = 74

^c^Defined as daily pain in bones, muscles, tendons, or ligaments for over 6 months

Of the 75 study participants, 57 (76%) were reached by phone 1 month following the ED visit to complete a follow-up assessment. Follow-up rates for the usual care, video only, and video plus telecare groups were 68%, 76%, and 84%, respectively. Of the 18 patients lost to follow up, 12 (67%) were due to phone numbers that were either disconnected or for which the phone rang but there was no response and no option for leaving a message. Excluding these patients, following the methods suggested by Sun et al. to call the number in the ED, would have produced a follow-up rate of 90% (57 patients out of 63), with rates for usual care, video only, and video plus telecare being 85%, 90%, and 95%, respectively.

Of the 50 patients that were randomized to receive the video, 46 (92%) completed the video. Of the 25 patients randomized to the video plus telecare group, 23 out of 25 patients were reached for the 48–72 hour follow-up call and 20 met criteria for a physician conversation. The most common criteria identifying patients at need for a physician conversation were a pain score of 4 or more (*n* = 12), pain affecting normal activities (*n* = 10), and pain affecting sleep (*n* = 9).

The changes in average pain from prior to the ED visit to 1 month after the ED visit for usual care, video only, and video plus telecare groups were -1.5, -2.2, and -3.0, respectively (Table 2). After adjusting for age, gender, and baseline pain severity, the change in pain for the usual care, video only, and video plus telecare groups was -1.3, -2.4, and -3.0. In the pairwise comparison analysis between intervention groups, the video plus telecare group had a 1.7-point (95% CI 1.2–2.1) greater decrease in pain compared to usual care, and the video alone had a 1.1-point (95% CI 0.6–1.6) greater decrease in pain compared to usual care after adjustment for baseline pain, age, and gender (Table 3). The percentage of patients reporting at least one medication side effect at 1 month for usual care, video only, and video plus telecare groups were 71%, 47%, and 38% respectively. Physical function scores at 1 month for usual care, video only, and video plus telecare groups were 2.8, 4.3, and 5.2.

**Table 2:** Outcomes at one month, intention-to-treat analysis (*n* = 57).

Outcome	Usual care (*n* = 17)	Video only (*n* = 19)	Video + telecare (*n* = 21)
Pain			
Baseline pain (SD)^a^	7.3 (2.3)	7.1 (2.7)	7.5 (2.0)
1-month pain (SD)^b^	5.8 (2.8)	4.9 (3.5)	4.5 (2.9)
Change in pain (SD)^c^	−1.3 (2.9)	−2.4 (3.2)	−3.0 (2.6)
Clinically significant decrease in pain, *n* (%)^d^	12 (71)	14 (74)	18 (86)
Physical function^e^			
Walking ability (SD)	0.9 (1.1)	1.4 (1.7)	1.4 (1.7)
Climbing ability (SD)	0.9 (1.1)	1.4 (1.6)	1.7 (1.5)
Carrying ability (SD)	1.1 (1.4)	1.4 (1.3)	2.1 (1.7)
Summary score of physical function (SD)	2.8 (2.8)	4.3 (4.0)	5.2 (3.9)
Total hours of sleep (SD)	5.8 (1.9)	6.0 (1.6)	6.0 (2.1)
Return to ED within 1 month, *n* (%)	2 (12)	3 (16)	2 (10)
PCP visit within 1 month, *n* (%)	12 (71)	11 (58)	16 (77)
Medication side effects, *n* (%)	12 (71)	9 (47)	8 (38)
New opioid prescribed after ED, *n* (%)	4 (24)	0 (0)	1 (5)

*CI* confidence interval, *ED* emergency department, *PCP* primary care provider

^a^Determined using 0–10 numerical rating scale to answer the question “Since your pain began, on average how intense has this pain been on a scale of 0–10, where 0 means no pain and 10 means pain as severe as it could possibly be?”

^b^Determined using 0–10 numerical rating scale to answer the question “What is the average amount of pain you have experienced over the last week on a scale of 0–10, where 0 means no pain and 10 means pain as severe as it could possibly be?”

^c^Adjusted for age, gender, and baseline pain severity. Unadjusted values for the three arms are −1.5, 2.2, and 3.0.

^d^Defined as a 1 point or more decrease in pain

^e^*N* = 53. Determined using a score from 0 to 12 using six self-reported questions about ability to walk, climb stairs, and lift things

**Table 3:** Difference in outcomes between randomization groups, adjusted for baseline pain, age, and gender

	Video + telecare vs. usual care	Video only vs. usual care	Video + telecare vs. video only
	Difference (95% CI)	Difference (95% CI)	Difference (95% CI)
Change in average pain	−1.7 (−1.2, −2.1)	−1.1 (−0.6, −1.6)	−0.6 (−0.1, −1.0)
Clinically significant decrease in pain (%)^a^	21 (−9,49)	13 (−19,43)	8 (−17,34)
Summary score of physical function	1.9 (1.3,2.4)	1.3 (0.7,1.8)	0.6 (0.0,1.2)
Mean hours of sleep	0.2 (−0.1, 0.5)	0.3 (0.0, 0.6)	0.1 (−0.2, 0.4)

^a^Defined as decrease in pain ≥1

Of the patients who completed follow up at 1 month, 68% reported following up with their primary care provider after their ED visit. ED pain medication prescriptions and recommendations for acetaminophen, NSAIDs, or opioids were similar in each of the study arms (Table 4). At 1 month, 21% of individuals who received usual care had been prescribed opioids by another physician following their ED visit versus 0% of patients who received the video only and 5% of those who received the video plus telecare.

**Table 4:** Pain medications by intervention group (*n* = 57)

	Usual care (*n* = 17)	Video only (*n* = 19)	Video + telecare (*n* = 21)
Prescription from ED	*n* (%)	*n* (%)	*n* (%)
Opioid	12 (71)	9 (47)	15 (71)
NSAID	1 (6)	2 (11)	6 (29)
Acetaminophen	5 (29)	3 (16)	6 (29)
New meds after the ED			
Opioid	4 (21)	0 (0)	1 (5)
NSAID	1 (6)	4 (21)	3 (14)
Acetaminophen	4 (24)	4 (21)	3 (14)

*ED* emergency department, *NSAID* nonsteroidal anti-inflammatory drug

## Discussion

We present a novel approach to improving pain outcomes for older patients presenting to the ED with musculoskeletal pain. The first component of the intervention is an interactive educational intervention designed to promote shared decision-making between the patient and the emergency provider and improved decision making following the ED visit by the patient. The second component is a telecare call at 48–72 hours which is scripted to support shared decision-making to identify changes in medications and behaviors to promote recovery.

Results of the trial suggest the potential for clinically important improvements in outcomes resulting from the intervention. Of the three intervention groups, the video plus follow-up phone call group experienced the greatest reduction in pain score at 1 month. Specifically, patients randomized to the full intervention of video plus telecare had a 3-point decrease in average pain during the first month following the ED visit, which was substantially more than the 1.5-point decrease observed in patients receiving usual care. Other outcomes favoring the video plus telecare group included better physical function, fewer side effects, and fewer patients received a prescription for an opioid from another provider. For most of these outcomes, results for patients randomized to the video alone were intermediate between the full intervention and usual care, suggesting some benefit from the video and an added benefit from the follow-up phone call. A larger trial is needed to confirm these findings. Additionally, 79% of participants were discharged from the ED and nearly one third of these did not follow up with a primary care physician despite having persistent pain symptoms. This is consistent with previous estimates [29], and supports the decision to study an ED-based intervention. Results of the pilot also indicate that fidelity to the intervention can be achieved based on high rates of completion of viewing the video and receipt of the telecare interventions among study participants randomized to these interventions.

This pilot study also identifies several challenges to the feasibility of conducting a clinical trial to improve the early management of acute musculoskeletal pain in older ED patients, which will inform the planning of a larger trial. Specific issues identified were irregular enrollment rates, low follow-up rates, and differential follow-up rates among the intervention groups.

One challenge to the feasibility of a large trial is the rate of participant enrollment. The target sample size for this study was approximately 25 patients in each intervention arm. This was achieved during an enrollment period of 11 months. However, because screening and enrollment was performed by volunteer RAs, for many weeks enrollment only occurred 1 or 2 days out of the week and only at one point during the day. This would not be the case with a large, funded study with a dedicated RA or assistants. It is our experience that when screening occurs every day, we identify 8–10 eligible patients a week. The consent rate among eligible patients of 77% is encouraging.

Another challenge identified in this pilot study was loss to follow up. The follow-up rate for this study was 76% and was differential across intervention groups. This is substantially lower than the 6-week follow-up rate of 93% in a large multicenter cohort by our research group [4]. One important difference between the pilot study and the large multicenter study is that patients received financial compensation for participation in the latter study but not the former. In reviewing reasons for loss to follow up within this pilot study, we see that non-working and unconfirmed phone numbers accounted for non-contact with 12 participants. A recently published description of methods to maximize follow up for ED research recommends trying patients’ phone numbers in the ED (in the patients presence) to confirm that the number works as an eligibility criterion [30]. If patients with the unconfirmed numbers were removed from the study sample, the follow-up rate would have changed to 90% (57 out of 63 patients).

Several other issues identified by the research team warrant further consideration for a larger trial. Because inpatient care is fundamentally different to outpatient care (e.g., patients have more serious pathology, have longer recovery times, and have a lot more directed medical care following watching the video in the ED), an ideal trial would only include discharged patients. However, the optimal time during the ED visit to enroll patients in a study and show them a video is while they are waiting for the results of tests and a decision on their disposition, not after they have been discharged. In a subsequent trial, additional criteria might be used to minimize the number of admitted patients who go through randomization.

We included a video-only arm in the trial because educational videos require fewer human resources than telecare and are likely to be easier to implement widely. The preliminary findings from this pilot trial suggest that the combined effect of the educational video plus telecare may have a greater effect on outcomes than the video alone. We plan to study video plus telecare and video alone again in a larger trial. No attention control was included in the usual-care arm because the intent of the large study will be to determine if the intervention improve outcomes when compared to what is usually done for these patients [31]. We recognize that the video or telecare may have therapeutic value apart from the specific content shared with the patient, and would like to estimate the effect of the intervention including these attention/other affects.

For the primary outcome for this study, we used change from average pain prior to the ED visit to the average pain in the past week at 1 month follow-up. Average pain prior to the ED visit is a preferable measure of baseline pain than current pain in the ED because some patients will have received analgesics prior to the ED interview. A composite outcome measure of pain symptoms based on current pain and average, maximum, and minimum pain in the past week or a combination of pain and interference with enjoyment of life due to pain and with general activity may provide a more comprehensive characterization of the burden of pain experienced at 1 month [32, 33].

The intervention may improve outcomes in several ways. First, the video may help prepare patients for informed conversations with emergency providers in the ED prior to discharge from them. In theory, this should enhance shared decision-making between patients and providers, an approach that has been associated with improved outcomes in this setting in observational studies [24, 25]. The value of the video may also extend beyond the ED by helping the patient to know how to dose medications, avoid taking multiple medications in the same class, prevent or recognize a side effect or adverse event, or increase recovery-promoting behaviors. Telecare may provide additional benefits to patients by creating the opportunity to optimize pain medication usage and address side effects. The telecare program is also designed to assess and encourage non-pharmacological strategies to manage pain. Although this pilot study gives some information on medication usage and behavior at follow up, the sample size and data collected in this pilot study are insufficient to properly examine the mechanisms underlying the effect of the intervention.

The intervention embraces two components identified as important in the ED discharge process in a recent report funded by the Agency for Healthcare Research and Quality: education and telephone follow up [34]. Other components identified in this report but not included in the intervention include ED-made follow-up appointments, prescription assistance, transportation assistance, and care coordination. Of these additional components, care coordination via electronic communication to the patient’s primary provider may be particularly valuable in this context and could be added as a third component of the intervention. The second component of the intervention was performed by a physician, but due to the cost of this resource, telecare provided by nurse care managers is a more common approach both in research and clinical practice and probably has greater potential for widespread implementation [35, 36].

There are several limitations to this study. A small sample size limits our ability to draw conclusions on the effect of the intervention. We did not collect information on health literacy. Although the video is designed to be informative across a broad range of health literacy, the effect of the intervention may differ by health literacy status [37]. We also did not collect information or examine for a differential effect among patients using opioids on a daily basis prior to the ED visit. Our clinical experience is that conversations about pain management with patients who routinely use opioids often are focused on the type and amount of opioid that is or is not going to be prescribed, which is different to conversations with patients who do not routinely use opioids. Future work may benefit from a distinct intervention for ED patients presenting with pain who are already using opioids on a daily basis.

## Conclusion

Findings from this pilot trial provide preliminary evidence of a possible benefit of an interactive video plus telecare intervention for older adults presenting to the ED with acute musculoskeletal pain. Results also indicate problems with follow up and suggest the need for adopting more aggressive methods for ensuring study participants can be reached at 1 month. A larger trial is planned to assess the efficacy of the intervention overall and in clinically important patient subgroups.

## Additional file

Additional file 1: Telecare protocol. (DOCX 147 kb)

## Abbreviations

ED: Emergency department
NSAID: Nonsteroidal anti-inflammatory drug
RA: Research assistant
