# Supplementary material for: Randomized controlled pilot study of an educational video plus telecare for the early outpatient management of musculoskeletal pain among older emergency department patients
Source: Trials. 2018 Jan 5;19:10. doi: 10.1186/s13063-017-2403-8 (PMC5756407; doi:10.1186/s13063-017-2403-8)
Supplement: Additional file 1: — Telecare protocol. (DOCX 147 kb) [file 13063_2017_2403_MOESM1_ESM.docx]

**Patient Assessment**

What is the patient’s pain score?

≥4

0-3

This patient will require a call from the study physician

Is this patient experiencing pain that is preventing them from doing activities that are important to them?

This patient will require a call from the study physician

This patient will require a call from the study physician

Yes

Does this patient wish to speak to a physician at this time for any reason?

This patient will require a call from the study physician

Yes

Yes

Is this patient experiencing side effects from pain medication?

This patient will require a call from the study physician

Yes

Is this patient experiencing pain that is affecting their sleep?

**Goals/Priorities Assessment**

What is the patient’s main goal for pain management?

Assess when pain is better/worse, discuss current medications, adjust dosage/timing, discuss alternative strategies

Reduce pain intensity

Improve function

- Take pain meds before activity
- Suggest physical activity, physical therapy, etc
- Intensive meds review, adjust dosage/timing

Minimize side effects

Improve enjoyment

- Discuss mood
- Social support

Find diagnosis

- Encourage follow-up with PCP

**Pharmacologic Recommendations**

What pain medication is this patient taking?

Recommend acetaminophen 650mg TID (BID if the patient has liver disease).

None

- If under-dosed, recommend acetaminophen 650mg TID (BID if the patient has liver disease).
- If adequate dose and no contraindications to NSAIDs (i.e. stomach ulcers, chronic renal disease, CHF, or on hypertension medication) recommend Naproxen 220mg BID for 5 days.
- If adequate dose and NSAID contraindications, recommend PMD appointment for opioid prescription.

Acetaminophen only

- Review opioid side effects.
- If patient is tolerating their medication, ensure that patient is taking maximum prescribed dose of opioid.
- Add acetaminophen 650mg TID (BID if the patient has liver disease) unless the patient is taking a combined opioid/acetaminophen.
- If patient is on max dose and still in pain, have them follow-up with PMD.
- Review NSAID dosing and continue if no side effects and not contraindicated.
- If under-dosed, recommend acetaminophen 650mg TID (BID if the patient has liver disease).
- If adequate dose, recommend PMD appointment for opioid prescription.
- Review NSAID dosing and continue if no side effects and not contraindicated.
- Recommend acetaminophen 650mg TID (BID if the patient has liver disease).

Opioid

NSAID only

Both acetaminophen and an NSAID

**Non-Pharmacologic Recommendations**

Is the patient sleeping at least seven hours per night?

- Reduce caffeine
- Go to sleep earlier at night
- Take pain medication before bed

No

Yes

Is the patient receiving emotional support from loved ones to help them deal with their pain?

- Communicate with loved ones
- Seek support for pain

No

Yes

- Recommend/adjust medications if necessary
- F/u w/ PCP

Is the patient experiencing anxiety or depression that makes it difficult to relieve their pain?

No

Yes

- Increase walking
- Strengthening exercises
- PT/OT

Has the patient reduced their physical activity or spent ½ day in bed since discharge from the ED?

No

Yes

Recommend follow up with primary care provider or UNC clinic for all patients not experiencing adequate pain relief.

UNC Family Medicine: 919-966-0210

UNC Internal Medicine: 919-966-1459

UNC Geriatrics: 919-957-6599

UNC SCHC: 919-843-6841
